# Self-care as a mediator between symptom-management self-efficacy and quality of life in women with breast cancer

**DOI:** 10.1371/journal.pone.0246430

**Published:** 2021-02-04

**Authors:** Chia-Hui Chin, Ling-Ming Tseng, Ta-Chung Chao, Tsae-Jyy Wang, Shu-Fang Wu, Shu-Yuan Liang

**Affiliations:** 1 Department of Nursing, Shin Kong Wu Ho-Su Memorial Hospital, Taipei, Taiwan; 2 Department of Medicine, Taipei Veterans General Hospital, Taipei, Taiwan; 3 College of Nursing, National Taipei University of Nursing and Health Sciences, Taipei, Taiwan; University of Central Florida, UNITED STATES

## Abstract

**Background:**

The important role of self-efficacy in facilitating health behavior and, in turn, promoting health outcomes has been widely presumed in the theoretical literature. However, little research has focused on the mechanism by which self-care mediates the relationship between symptom-management self-efficacy and quality of life (QOL) in breast cancer patients.

**Objective:**

The purpose of this study was to examine the relationship between symptom-management self-efficacy and quality of life in Taiwanese oncology outpatients with breast cancer and then proposes self-care as a mediator between these two factors.

**Methods:**

This cross-sectional study enrolled 201 oncology outpatients at one teaching hospital in metropolitan Taipei City, Taiwan. The research instruments included the Symptom-Management Self-Efficacy Scale—Cancer (SMSES-Breast Ca.), the Self-Care Scale, and the European Organization for Research & Treatment of Cancer Quality of Life Group Questionnaire (EORTC-QLQ-C30).

**Results:**

Symptom-management self-efficacy (SMSE) was directly associated with the QOL of the participants (β = 5.94, *p* < .001). Moreover, SMSE was indirectly associated with QOL through self-care. Self-care was found to mediate the relationship between symptom-management self-efficacy and global QOL (indirect effect = 0.54, 95% CI 0.12 to 1.18). The level of 95% CI was significant.

**Conclusions:**

The present study supports that self-efficacy beliefs and self-care both significantly and positively influence the quality of life of patients.

## Introduction

Quality of life is an important index for evaluating the outcomes of treatment and the long-term survival of patients with cancer [1, 2]. In light of the limitations of current medical science, the goal of cancer treatment is controlling symptoms and delaying disease progression rather than curing the disease. Therefore, quality of life is a crucial determinate of symptom relief and of the rehabilitation needs of patients with cancer.

Quality of life assessments are increasingly used in the care of patients with cancer. Furthermore, quality of life is a particularly important outcome indicator for patients with advanced breast cancer who receive chemotherapy [3–5], as this treatment strategy causes side effects such as gastrointestinal problems and neurological disorders that not only impact significantly upon quality of life [3, 4] but also predict the premature discontinuation of treatment [6].

Promoting and maintaining the self-care behavior that is necessary to manage the side effects of chemotherapy and to improve quality of life are important for patients with cancer [7, 8]. However, given the challenging nature of performing self-care activities while dealing with the side effects of chemotherapy [3, 9, 10], it is critical to understand the factors and mechanisms that help predict the self-care behavior and quality of life of patients.

Social and behavioral scientists have proposed that a person’s belief system crucially influences his or her behavior and, therefore, the related health outcomes [11–13]. For example, studies suggest that beliefs regarding pharmacological strategy such as opioid-taking self-efficacy influence a person’s ability to adhere to medical prescriptions for cancer pain and thus affect pain-control outcomes [14]. Moreover, the beliefs regarding self-managing the side effects of chemotherapy that are held by patients with breast cancer may influence their self-care behavior and their quality of life. Thus, the purposes of the present study were to: 1) examine the association between symptom-management self-efficacy and quality of life and 2) assess the role of self-care as a mediator between symptom-management self-efficacy and quality of life in Taiwanese oncology outpatients with breast cancer.

## Methods

Institutional review board: Taipei Veterans General Hospital (2011-03-052IC#1) approved this research. The written consent form was obtained from participant.

### Sample and procedure

The present study used a convenience sample of 201 women. All eligible breast cancer patients who were admitted to the outpatient oncology unit of one teaching hospital in metropolitan Taipei City during the period of data collection were invited to participate. The eligibility criteria for participation included: (1) having undergone three cycles of chemotherapy for the treatment of breast cancer; (2) older than 18 years of age; and (3) conscious and willing to sign informed consent.

The ethics committee of the hospital approved the research plan for the present study. All of the patients who expressed interest in participating were asked to sign an informed consent form prior to completing the research questionnaire. The study questionnaire packet was then provided to the participants. After participants had completed all of the questionnaires, the researcher reviewed each questionnaire for missing data. In the event of missing data, the researcher contacted the relevant participants to ensure that all required data was provided. Data on the medical characteristics of participants were collected from hospital medical records.

### Measures

#### Sociodemographic variables

The sociodemographic data on participants that were collected for the present study included: age, education, household income, religion, marital status, employment status, and living status. The medical data that were collected included: cancer stage, time since diagnosis, metastases, and surgery status.

#### Symptom-Management Self-Efficacy (SMSE)

The Symptom-Management Self-Efficacy Scale—Breast Cancer (SMSES-Breast Ca.) was used to assess symptom-management self-efficacy [15]. This scale includes three subscales, with a total of 27 items that measure perceived self-efficacy in terms of the respondent’s ability to self-manage the symptoms of chemotherapy. The subscales of the SMSES-Breast Ca. are: acquiring problem-solving (7 items), managing chemotherapy-related symptoms (15 items), and managing emotional and interpersonal disturbance (5 items). Internal consistency values (Cronbach’s alpha) for the three subscales and for the total scale were: .88 for acquiring problem-solving, .95 for managing chemotherapy-related symptoms, .88 for managing emotional and interpersonal disturbance, and .96 for the total scale [15]. The test-retest coefficient (2-week interval) of the SMSES ranged from 0.40 to 0.78 [15]. An 11-point Likert scale was used for each item that ranged from 0 (not confident at all) to 10 (completely confident), with higher scores representing higher perceived self-efficacy.

#### Self-care

The Self-Care Scale was used in the present study to measure the self-care activities of participants. This scale was developed and validated by Yang [16] with a Cronbach’s alpha of 0.83 for reliability. The instrument is self-reported and rated on a 4-point Likert scale, with 0 representing “never” and 3 representing “always”. Some examples of the items are as follows: “Brush the teeth using a soft toothbrush,” “Clean the perineum after going to toilet,” and “Wear a hat when going out”. Higher scores for the scale indicate that the respondent engages in a greater number of self-care activities in order to manage symptoms.

#### Quality of life (QOL)

Quality of life was measured using the global QOL of the European Organization for Research and Treatment of Cancer Quality of Life Group Questionnaire (EORTC QLQ-C30 version 3.0). The EORTC QLQ-C30 contains two items for a global QOL. The items on this scale are scored from 1 to 7, with 1 indicating ‘very poor’ and 7 indicating ‘excellent’. For the purposes of the present study, scoring was converted to a 0-100-point scale, with higher scores associated with more positive global QOL.

The EORTC QLQ-C30 is a widely used questionnaire for assessing quality of life in cancer patients. This questionnaire has been validated in multiple countries for various cancer diagnoses [17–20] and a validated Chinese version is currently available [21].

### Statistical analysis

**Fig 1:**
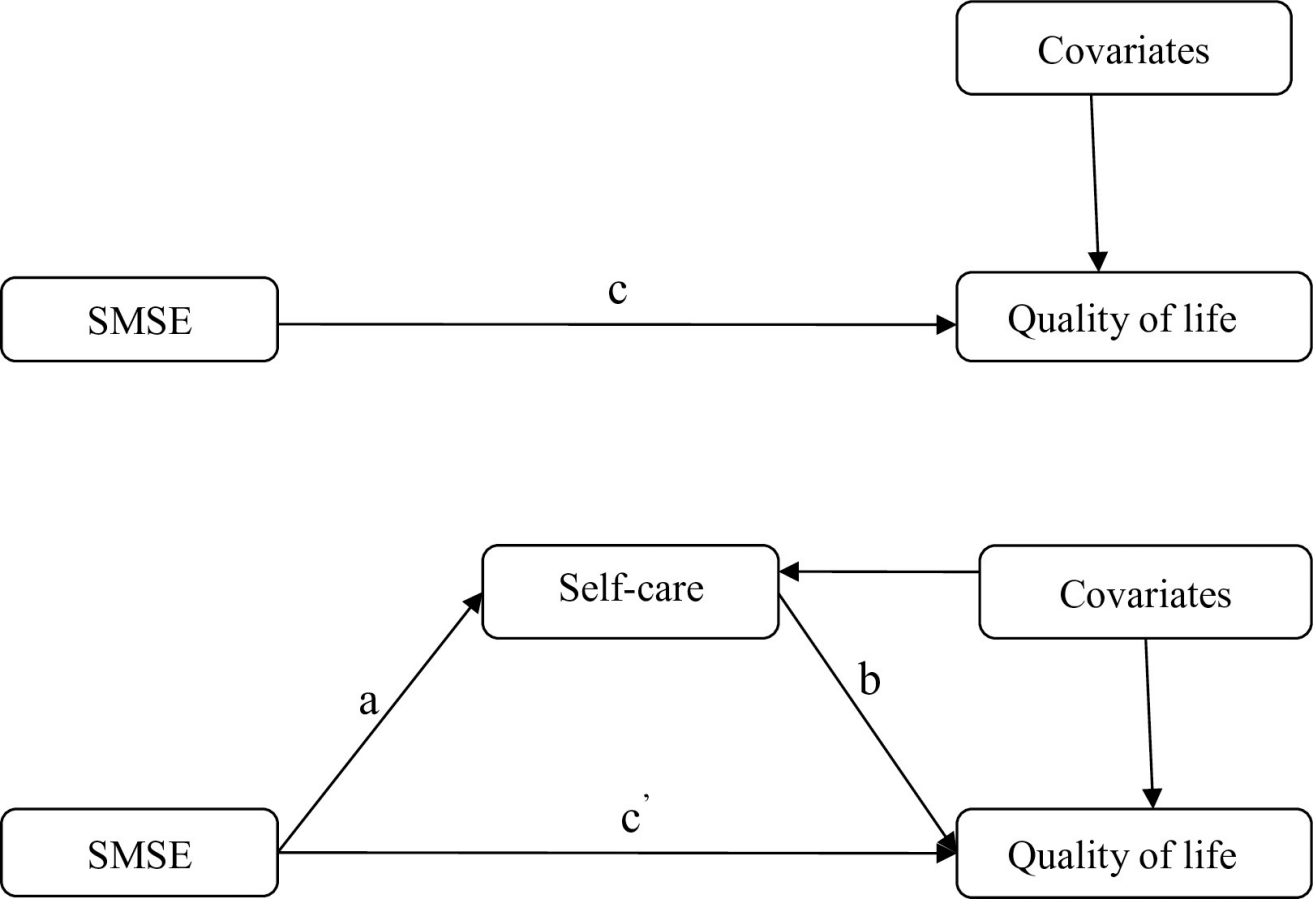
Graphic representation of the proposed mediation model.

The bivariate relationships among the main study variables were examined using Pearson’s correlation. The mediation analyses were evaluated using two approaches: the linear regression analyses of Baron and Kenny [22] and the bootstrapped method of Shrout and Bolger [23]. The former requires that three conditions must be met: (1) the direct effect of an independent variable on a dependent variable (*c* path) is significant, (2) the effect of an independent variable on a mediator (*a* path) is significant, and (3) the effect of a mediator on a dependent variable (*b* path) is significant while accounting for the independent variable (shown in Fig 1).

Moreover, the bootstrapping method was used to verify the mediation effect in accordance with the procedures of Shrout and Bolger [23]. A total of 1,000 bootstrapped samples were generated, for which point estimates and 95% bias-corrected and accelerated confidence intervals for indirect effects (*a* × *b*) were calculated. The mediation analyses were performed using the “PROCESS program” on SPSS v22 (IBM SPSS Inc, Chicago, Illinois), with adjustments made for the control variables. A mediated effect was determined when the bootstrapped 95% confidence intervals of the indirect effect did not include zero. In the aforementioned analyses, all of the demographic and medical variables were introduced into the model as covariates (control variables).

## Results

### Demographic and clinical characteristics of the participants

The mean years of age and education of participants were 53.6 (SD = 9.5 years, range: 32–80 years) and 12.5 (SD = 4.1 years, range: 0–24 years). A majority of participants were unemployed (73.6%), married (70.6%), living with others (75.1%), and affiliated with a religion (83.6%). In addition, participants had a mean duration of breast cancer of 4.2 years (SD = 5.4 years, range: 0.17–57.08 years). The majority of subjects experienced tumour stage 1–2 (48.8%), underwent surgery for their cancer (92%), and had received a diagnosis of a metastatic malignancy (64.7%). Full details of the demographic and clinical characteristics of the participants for this study have been reported elsewhere [24].

### Correlation among the main study variables

Table 1 shows the bivariate correlations among the main study variables. SMSE and self-care both significantly and positively correlated to the global QOL (*r* = .50, *p* < .001; *r* = .27, *p* < .001, respectively). Moreover, SMES correlated significantly with self-care (*r* = .30, *p* < .001).

**Table 1:** Correlation among the main variables of interest (*N* = 201).

Variable	Mean ± *SD*	1	2	3
1. SMSE	7.1 ± 1.9	—		
2. Self-care	1.9 ± 0.5	.30***	—	
3. QOL	66.4 ± 21.8	.50***	.27***	—

***p < .001.

Abbreviations: QOL = quality of life; SMSE = symptom-management self-efficacy.

### Mediation analyses

Table 2 shows the results for the potential mediating effect. The total effect of SMSE on QOL (path *c*) was tested in Model 1, with adjustments made for other control variables (covariates). Model 2 evaluated the association between SMSE and self-care (path *a*). Finally, Model 3 assessed the direct effect of self-care on QOL after controlling for SMSE and the control variables (covariates; path *b*).

**Table 2:** Tests of the potential mediating effect (multiple linear regression analysis).

	Model 1:		Model 2:		Model 3:	
	QOL		Self-care		QOL	
Variable	*β*	*t*	*β*	*t*	*β*	*t*
Intercept	52.33	3.62***	1.01	2.74**	45.51	3.12**
Age (by decade)	0.56	0.33	0.01	0.11	0.53	0.31
Education (by years of schooling)	-0.89	-2.29*	0.01	1.17	-0.97	-2.51*
Years since diagnosis (log scale)	-4.47	-3.22**	-0.04	-1.24	-4.18	-3.03**
Income						
0.5~1 *vs*. below 0.5 million NTD	1.96	0.60	0.04	0.46	1.71	0.53
1~1.5 *vs*. below 0.5 million NTD	-6.09	-1.38	0.15	1.37	-7.14	-1.63
Above 1.5 *vs*. below 0.5 million NTD	1.59	0.36	-0.01	-0.11	1.67	0.38
Employment (yes *vs*. no)	6.91	2.10*	-0.11	-1.30	7.64	2.34*
Marital status						
Unmarried *vs*. married	-7.18	-1.72	0.01	0.06	-7.23	-1.76
Other *vs*. married	-1.86	-0.47	0.05	0.48	-2.19	-0.56
Living with someone (yes *vs*. no)	3.88	1.23	0.04	0.52	3.59	1.15
Religious affiliation (yes *vs*. no)	-7.51	-2.07*	-0.03	-0.34	-7.30	-2.04*
Stage						
1–2 *vs*. 0	-13.74	-1.96	-0.04	-0.20	-13.50	-1.95
3–4 *vs*. 0	-16.70	-2.35*	0.02	0.09	-16.81	-2.39*
Other *vs*. 0	-7.17	-0.94	-0.03	-0.17	-6.94	-0.92
Metastasis						
Yes *vs*. no	-3.31	-0.98	-0.04	-0.45	-3.05	-0.92
Unknown *vs*. no	-1.76	-0.33	-0.15	-1.07	-0.78	-0.15
Surgery (yes *vs*. no)	2.30	0.44	0.24	1.80	0.67	0.13
SMSE	5.94	8.23***	0.08	4.31***	5.41	7.22***
Self-care	—	—	—	—	6.76	2.35*
Model *F*	6.25***		1.77*		6.35***	
*R*^2^	38.2%		14.9%		40.0%	
Adjusted *R*^2^	32.1%		6.5%		33.7%	

*p < .05

**p < .01

***p < .001.

*Note*: β = unstandardized regression coefficient.

Abbreviations: QOL = quality of life; SMSE = symptom-management self-efficacy.

30 NTD (New Taiwan dollars) = US $1.

The results suggest the presence of significantly positive associations between SMSE and QOL (β = 5.94, *p* < .001, from Model 1) and between SMSE and self-care (β = 0.08, *p* < .001, from Model 2) as well as of an independent and significant association between increased self-care activities and higher QOL in the presence of SMSE (β = 6.76, *p* < .05, from Model 3). This result supports the argument for the mediating role of self-care.

**Fig 2:**
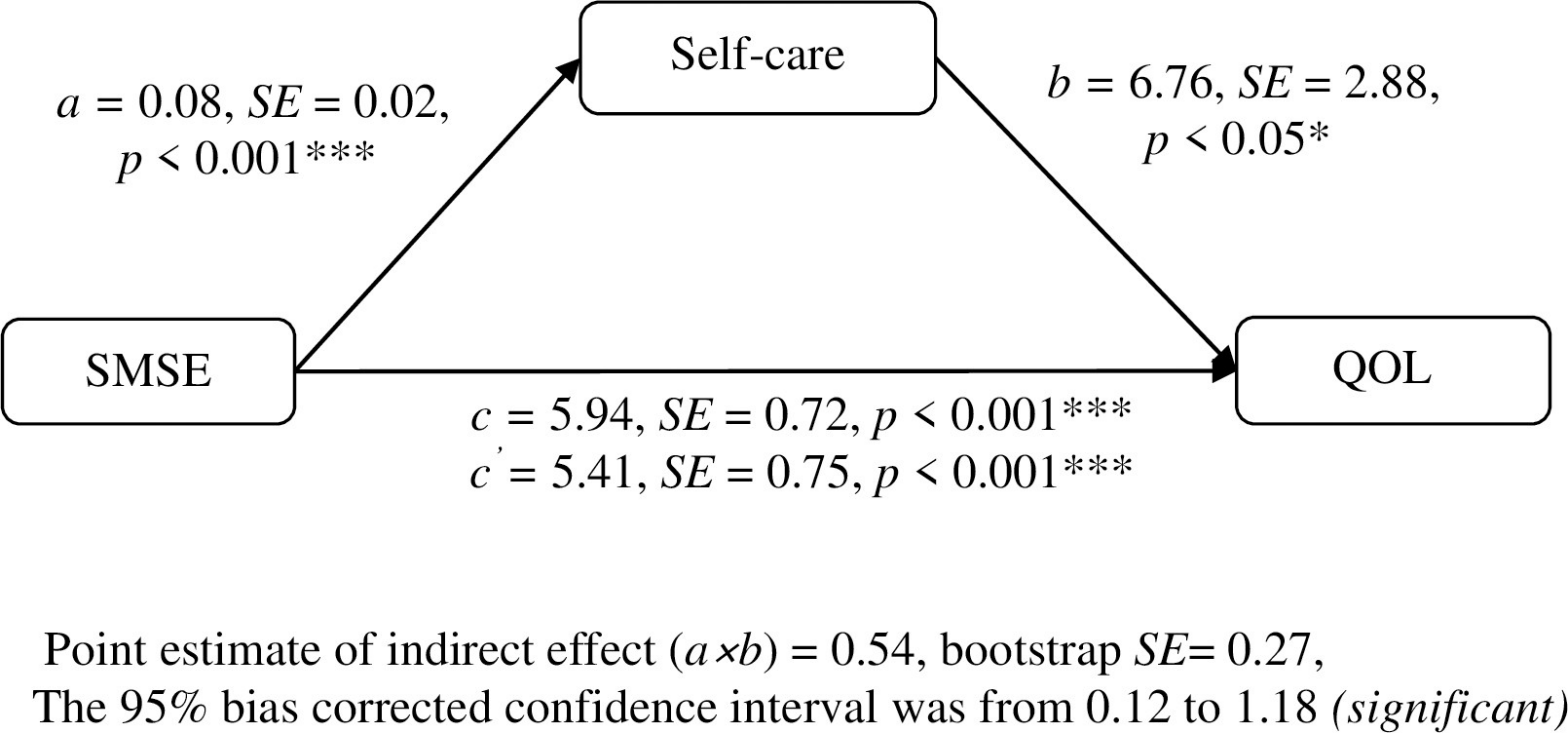
The mediating role of self-care in the relationship between SMSE and QOL. Abbreviations: SMSE = symptom-management self-efficacy; QOL = quality of life.

Fig 2 illustrates the mediation role of self-care in the association between SMSE and QOL as identified using the bootstrapping method. The result shows that the accelerated bootstrapping confidence intervals of the indirect effect did not include zero (range: 0.12 to 1.18), which verified the mediating effect of self-care in the relationship between SMSE and patient’s QOL.

In terms of the control variables (covariates), the result indicated that higher levels of education (β = -0.97, *p* < .05), longer duration since diagnosis (β = -4.18, *p* < .01), having a religion affiliation (β = -7.30, *p* < .05), and stage III/IV (β = -16.81, *p* < .05) were each significantly associated with worse QOL. Furthermore, participants who were employed had better QOL than their unemployed peers (β = 7.64, *p* < .05; Model 3 in Table 2)

## Discussion

To the knowledge of the authors, this is the first study to focus on the mediation effect of self-care on symptom-management self-efficacy and its effect on the quality of life of breast-cancer patients who are receiving chemotherapy. Although this effect has been widely assumed, it has not previously been established empirically. Understanding the explanatory mechanism of better self-care in the correlation between greater self-efficacy and higher QOL may assist clinicians, researchers, and theorists to better understand the mechanism by which self-efficacy affects health outcomes. The present study is important not only because the findings contribute to theoretical knowledge but also because it provides important inferences for the development of related interventions and clinical practices.

Consistent with prior research, the findings revealed that greater self-efficacy was associated significantly with better self-care [25, 26] and with higher patient QOL [27–29]. Furthermore, our finding that better self-care was significantly associated with higher patient QOL also echoed the results of prior research [30, 31].

Importantly, the results of the present study offer evidence supporting that the association between higher self-efficacy and greater QOL is mediated, or partially explained, by better self-care. Greater symptom-management self-efficacy works across better self-care, which, in turn, triggers higher QOL in patients. Self-efficacy is an active agent in patient health outcomes, with higher self-efficacy patients taking greater responsibility to adopt behaviors that address their chemotherapy-related symptoms and improve QOL [11, 32]. Therefore, self-efficacy was shown in the present study to influence the self-care behavior and the QOL of participants.

The present study has multiple implications for clinical practice. The mediative effect of self-care in the relationship between self-efficacy and quality of life suggests that interventions that aim to improve the QOL of cancer patients should focus on improving the symptom-management self-efficacy of patients, teaching relevant knowledge and skills, and coaching on approaches to manage chemotherapy-related symptoms [31, 32]. In summary, the results of the present study suggest that facilitating self-care behaviors will have a significantly greater positive effect on the QOL of patients when this facilitation is combined with interventions that improve the symptom-management self-efficacy of patients.

Bandura’s social cognitive theory provides a helpful basis for reflecting how an intervention may be used to improve a patient’s symptom-management self-efficacy. Particularly, recognizing and emphasizing patients’ existing and previous successes in managing their chemotherapy-related symptoms will help create the optimistic reinforcement that is critical to building self-efficacy. In addition, breast cancer patients who received chemotherapy may benefit from attending social support groups in terms of enhanced self-efficacy sensitivities and social modeling capabilities. A recent work by Weber et al. demonstrated that involvement in a dyadic support-group program improved self-efficacy in cancer patients [33]. Furthermore, using demonstrations that employ various materials combining pictures, drawings, and/or actors of different ages, genders, and body types has been shown to be effective in enhancing symptom management behavior. Bandura’s work also proposes social persuasion as a useful tool in improving self-efficacy. Breast cancer outpatients that have received chemotherapy may attain better self-care behaviors and greater QOL if clinicians enhance the positive symptom-management self-belief of these outpatients by using persuasive, credible communication.

Huang et al. [34] used a self-efficacy enhancing program focused on setting achievable goals and actions for existing successes, sharing successful stories for vicarious experiences, providing positive feedbacks for verbal persuasion, and incorporating social support for social modeling to improving quality of life of patients with cancer.

Researchers suggested a self-care program to improving cancer patients’ self-care and quality of life [31]. This program involved a two-person team including a nurse with field experience and a healthcare professional for social support. Key content areas of this program for symptom self-care included exercise for fatigue, and care activity for skin, diet, rest and sleep related to chemotherapy. This program significantly improved the quality of life of patients with cancer [31].

Health professionals may incorporate strategies focused on improving patients’ symptom-management self-efficacy and self-care behavior to improve their quality of life. Particularly, self-care plays an important mechanism by which symptom-management self-efficacy influencing patients’ quality of life.

There are several important limitations to the present study. The study used a cross-sectional sample, which may be subject to a causal effect after the mediation tests. Thus, similar studies that employ a longitudinal approach to further investigate the proposed mediating model are necessary. In addition, our use of a convenience sample and mix of patients with various stages may mean that results are not generalizable to the wider population of breast cancer patients. Replicating this study in larger groups that represent broader demographic parameters and a wide variety of hospital settings will be necessary in order to ensure that findings may be generalized to the general population.

## Supporting information

S1 File (PDF)

S2 File (PDF)

S3 File (SAV)
