# Supplementary material for: Self-care as a mediator between symptom-management self-efficacy and quality of life in women with breast cancer
Source: PLoS One. 2021 Feb 4;16(2):e0246430. doi: 10.1371/journal.pone.0246430 (PMC7861359; doi:10.1371/journal.pone.0246430)
Supplement: S1 File — (PDF) [file pone.0246430.s001.pdf]

Symptom-Management Self-Efficacy Scale - Breast Cancer (SMSES-BC)

The Symptom-Management Self-Efficacy Scale-Breast Cancer (SMSES-BC) is a 27-item questionnaire used to assess the multidimensional nature of self-efficacy in relation to symptom management relevant to chemotherapy for patients with breast cancer. Items are rated using a scale ranging 0 to 10, with 0 indicating “not at all confident” and 10 indicating “completely confident”. According to subjects’ responses on the 11-point scale, a total score and summarised total scores for subscales can be obtained. A higher score means higher perceived symptom-management self-efficacy.

The construct validity of the SMSES-BC was assessed by Liang, Wu, Kuo, and Lu [1]. Factor analysis confirmed a three factor structure, with a coefficient alpha of 0.96 for the total scale and ranging from 0.88 to 0.95 for the three subscales emerging from this analysis. The three factor structure results in the following subscales: acquiring problem-solving (7 items), managing chemotherapy-related symptoms (15 items), and managing emotional and interpersonal disturbance (5 items). Test-retest reliability of the subscales in an earlier pilot study was from 0.40 to 0.78 ($p < .01$) over a 2-week period.

1. Liang, S. Y., Wu, W. W., Kuo, C. Y., & Lu, Y. Y. (2015). Development and preliminary evaluation of psychometric properties of the Symptom-Management Self-Efficacy Scale for breast cancer. *Asian Nursing Research*, 9(4), 312-317. 10.1016/j.anr.2015.09.001

Symptom-Management Self-Efficacy Scale - Breast Cancer (SMSES-BC)

Below is a list of activities you may have to perform when taking care yourself in relation to symptom management relevant to chemotherapy. Please read each question and circle the number that best describes how confidently you can perform each activity, even in situations which you have not directly experienced.

**Items**<br>***I am confident that…***		***Not at all confident***										***Completely confident***
1	Managing social activity disruptions (e.g., stopping gathering, stopping gossip)	0	1	2	3	4	5	6	7	8	9	10
2	Managing emotional distress (e.g., feeling down, powerless, worry, fear)	0	1	2	3	4	5	6	7	8	9	10
3	Managing palpitations (e.g., tachycardia)	0	1	2	3	4	5	6	7	8	9	10
4	Managing fatigue (e.g., tiredness, weakness)	0	1	2	3	4	5	6	7	8	9	10
5	Managing interpersonal stress (e.g., stress from people paying attention)	0	1	2	3	4	5	6	7	8	9	10
6	Managing nausea and vomiting	0	1	2	3	4	5	6	7	8	9	10
7	Managing endocrine problems (e.g., night sweat, flush)	0	1	2	3	4	5	6	7	8	9	10
8	Seeking place for emotion (e.g., religion, painting, patchwork, book)	0	1	2	3	4	5	6	7	8	9	10
9	Actively talk with health professional about the side effects of chemotherapy before treatment	0	1	2	3	4	5	6	7	8	9	10
10	Managing the problems related to oral mucosa (e.g., mucositis, cheilosis)	0	1	2	3	4	5	6	7	8	9	10
11	Actively talk with health professional about my side effects of chemotherapy after treatment	0	1	2	3	4	5	6	7	8	9	10
12	Managing sleeping problems (e.g., insomnia, light sleeping)	0	1	2	3	4	5	6	7	8	9	10
13	Managing eating problems (e.g., difficulty in swallowing, parageusia, poor appetite)	0	1	2	3	4	5	6	7	8	9	10
14	Managing skin problems (e.g., darkening, decortication, skin rash, itching)	0	1	2	3	4	5	6	7	8	9	10
15	Preventing infection (e.g., anemia, blood cells decreasing)	0	1	2	3	4	5	6	7	8	9	10
16	Managing pain (e.g., bone pain, sore muscles, spasm)	0	1	2	3	4	5	6	7	8	9	10
17	Managing nail problems (e.g., darkening, deformation, burst)	0	1	2	3	4	5	6	7	8	9	10
18	Obtaining support from social group (e.g., peer group, church member)	0	1	2	3	4	5	6	7	8	9	10
19	Managing peripheral problems of limbs (e.g., numbness, rigid)	0	1	2	3	4	5	6	7	8	9	10
20	Actively talk with health professional about the management of side effects of	0	1	2	3	4	5	6	7	8	9	10

**Items**<br><br>***I am confident that…***		***Not at all confident***										***Completely confident***
	chemotherapy											
21	Managing memory problems (e.g., short memory, forgetful)	0	1	2	3	4	5	6	7	8	9	10
22	Obtaining internet resources to manage the problems related to chemotherapy	0	1	2	3	4	5	6	7	8	9	10
23	Managing hair loss	0	1	2	3	4	5	6	7	8	9	10
24	Managing interpersonal isolation	0	1	2	3	4	5	6	7	8	9	10
25	Managing the work problems related to chemotherapy (e.g., asking for sick leave)	0	1	2	3	4	5	6	7	8	9	10
26	Obtaining support from surrounding people (e.g., health professional, family, friend)	0	1	2	3	4	5	6	7	8	9	10
27	Managing gastrointestinal problems (e.g., distention, constipation, diarrhea)	0	1	2	3	4	5	6	7	8	9	10

Subscale Names and Item Descriptions for the SMSES-BC

Item
**Subscale 1 (7 items): Acquiring Problem-Solving (AP)**
9. Actively talk with health professional about the side effects of chemotherapy before treatment
11. Actively talk with health professional about my side effects of chemotherapy after treatment
18. Obtaining support from social group (e.g., peer group, church member)
20. Actively talk with health professional about the management of side effects of chemotherapy
22. Obtaining internet resources to manage the problems related to chemotherapy
25. Managing the work problems related to chemotherapy (e.g., asking for sick leave)
26. Obtaining support from surrounding people (e.g., health professional, family, friend)
**Subscale 2 (15 items): Managing Chemotherapy-Related Symptoms (MC)**
3. Managing palpitations (e.g., tachycardia)
4. Managing fatigue (e.g., tiredness, weakness)
6. Managing nausea and vomiting
7. Managing endocrine problems (e.g., night sweat, flush)
10. Managing the problems related to oral mucosa (e.g., mucositis, cheilosis)
12. Managing sleeping problems (e.g., insomnia, light sleeping)
13. Managing eating problems (e.g., difficulty in swallowing, parageusia, poor appetite)
14. Managing skin problems (e.g., darkening, decortication, skin rash, itching)
15. Preventing infection (e.g., anemia, blood cells decreasing)
16. Managing pain (e.g., bone pain, sore muscles, spasm)
17. Managing nail problems (e.g., darkening, deformation, burst)
19. Managing peripheral problems of limbs (e.g., numbness, rigid)
21. Managing memory problems (e.g., short memory, forgetful)
23. Managing hair loss
27. Managing gastrointestinal problems (e.g., distention, constipation, diarrhea)
**Subscale 3 (5 items): Managing Emotional and Interpersonal Disturbance (ME)**
1. Managing social activity disruptions (e.g., stopping gathering, stopping gossip)
2. Managing emotional distress (e.g., feeling down, powerless, worry, fear)
5. Managing interpersonal stress (e.g., stress from people paying attention)
8. Seeking place for emotion (e.g., religion, painting, patchwork, book)
24. Managing interpersonal isolation
