# Supplementary material for: Self-care as a mediator between symptom-management self-efficacy and quality of life in women with breast cancer
Source: PLoS One. 2021 Feb 4;16(2):e0246430. doi: 10.1371/journal.pone.0246430 (PMC7861359; doi:10.1371/journal.pone.0246430)
Supplement: S2 File — (PDF) [file pone.0246430.s002.pdf]

自我照顧量表

項目	從來沒有	有時如此	常常如此	一直如此
治療期間的健康照護：				
1.增加休息時間	0	1	2	3
2.盡可能維持日常的生活型態	0	1	2	3
3.依體力狀況計畫活動的程度，漸進式增加活動的量	0	1	2	3
4.感到疲倦時，將一些事委託家人或朋友代勞	0	1	2	3
5.避免曬太陽	0	1	2	3
6.皮膚乾燥時，使用水溶性的乳液	0	1	2	3
7.使用空調，注意室溫及溼氣的調節	0	1	2	3
8.使用溫水梳洗，避免水溫過冷或過燙	0	1	2	3
9.皮膚癢時以指腹按壓方式，不用手指抓	0	1	2	3
10.少量多餐，維持足夠的飲食攝入	0	1	2	3
11.嘗試不同類型的飲食，來促進食慾	0	1	2	3
12.吃高熱量、高蛋白的食物	0	1	2	3
13.每天喝 2000C.C.以上的水	0	1	2	3
14.經常漱口保持口腔濕潤	0	1	2	3
15.使用軟毛牙刷刷牙	0	1	2	3
16.吃溫和、質軟、不刺激口腔黏膜的食物	0	1	2	3
17.常潤滑嘴唇	0	1	2	3
18.不使用或小心使用牙線及牙籤	0	1	2	3
19.避免會造成身體損傷的活動，如搬運東西	0	1	2	3
20.刮鬍子或剃毛時使用電動刮鬍刀	0	1	2	3
21.不穿太緊或質料粗糙的衣服	0	1	2	3
22.治療期間避免出入公共場所	0	1	2	3
23.當家人有感冒情形時，避免接觸或戴口罩	0	1	2	3
24.如廁後做會陰部的清潔	0	1	2	3
25.每三到五天洗一次頭髮	0	1	2	3
26.用中性、無刺激性、含蛋白質的洗髮精洗頭	0	1	2	3
27.外出時，戴假髮或帽子	0	1	2	3
28.感到緊張時會利用深呼吸或其他放鬆的方法	0	1	2	3
29.睡不著覺時，會利用聽音樂、看書或其他方法來促進睡眠	0	1	2	3
30.只吃醫師開的藥，並且按時服用	0	1	2	3
31.按照時間定期至醫院做檢查及治療	0	1	2	3
